# Solar Wind Protons in the Diamagnetic Cavity at Comet 67P/Churyumov‐Gerasimenko

**DOI:** 10.1029/2022JA031249

**Published:** 2023-04-18

**Authors:** Charlotte Goetz, Lucie Scharré, Cyril Simon Wedlund, Anja Moeslinger, Hans Nilsson, Elias Odelstad, Matthew G. G. T. Taylor, Martin Volwerk

**Affiliations:** ^1^ Department of Mathematics, Physics and Electrical Engineering Northumbria University Newcastle‐upon‐Tyne UK; ^2^ European Space Research and Technology Centre European Space Agency Noordwijk The Netherlands; ^3^ Old College University of Edinburgh Edinburgh UK; ^4^ Institute for Physics, Laboratory for Galaxy Evolution and Spectral Modelling Ecole Polytechnique Federale de Lausanne, Observatoire de Sauverny Versoix Switzerland; ^5^ Space Research Institute Austrian Academy of Sciences Graz Austria; ^6^ Swedish Institute of Space Physics Kiruna Sweden; ^7^ Swedish Institute of Space Physics Uppsala Sweden

## Abstract

The plasma environment at a comet can be divided into different regions with distinct plasma characteristics. Two such regions are the solar wind ion cavity, which refers to the part of the outer coma that does not contain any solar wind ions anymore; and the diamagnetic cavity, which is the region of unmagnetized plasma in the innermost coma. From theory and previous observations, it was thought that under usual circumstances no solar wind ion should be observable near or inside of the diamagnetic cavity. For the first time, we report on five observations that show that protons near solar wind energies can also be found inside the diamagnetic cavity. We characterize these proton signatures, where and when they occur, and discuss possible mechanisms that could lead to protons penetrating the inner coma and traversing the diamagnetic cavity boundary. By understanding these observations, we hope to better understand the interaction region of the comet with the solar wind under nonstandard conditions. The protons detected inside the diamagnetic cavity have directions and energies consistent with protons of solar wind origin. The five events occur only at intermediate gas production rates and low cometocentric distances. Charge transfer reactions, high solar wind dynamic pressure and a neutral gas outburst can be ruled out as causes. We suggest that the anomalous appearance of protons in the diamagnetic cavity is due to a specific solar wind configuration where the solar wind velocity is parallel to the interplanetary magnetic field, thus inhibiting mass‐loading and deflection.

## Key Points

- Unexpectedly, protons of solar wind origin were observed in the diamagnetic cavity at comet 67P
- We have ruled out internal and external transients as well as charge‐exchange as triggers for this unusual observation
- We theorize protons are able to penetrate the diamagnetic cavity due to solar wind conditions with the velocity and magnetic field parallel

## 1 Introduction

Comets are small bodies that traverse the solar system on often highly elliptical orbits. Their nuclei are made up of loosely packed ice and dust (Blum et al., 2017) and are usually a couple of kilometers in diameter. As comets approach the Sun, the insolation will heat the surface and trigger sublimation of the ices. Because of their small gravitational pull, this gas can expand into the vacuum of space, where it is ionized by photoionization, electron impact ionization or charge‐exchange (Heritier et al., 2018). Since most of the ice on the surface is water or carbon dioxide, the plasma that is created consists mostly of water or carbon dioxide ions. These heavy ions are initially at rest in the cometary frame of reference and appear as an obstacle to the expanding solar wind. Their incorporation into the solar wind flow is an interesting plasma physical process that has raised many open questions since the beginning of the space age (see Szegö et al., 2000, and references therein).

The interaction of the solar wind with a comet can take many shapes and forms, depending on the activity of the comet. One of the most exciting phenomena is the formation of a diamagnetic cavity (DC), a region in the innermost plasma environment that is completely free of a magnetic field. Biermann et al. (1967) first speculated that such a region might exist with the help of a simple gas dynamic model of the solar wind and the flow around a comet. They found that the addition of heavy cometary ions to the solar wind flow (mass‐loading) would result in a reduction of the flow velocity up until a stagnation point. Since 67P, and presumably all other comets, are unmagnetized (Auster et al., 2015), the magnetic field that is observable near a comet is entirely of solar wind origin. Thus, in an MHD approximation, if the solar wind flow stops, this interplanetary magnetic field (IMF) cannot continue further and a field‐free region is created around the nucleus. The size of this region depends on the outgassing rate and solar wind conditions (Goetz et al., 2016a, 2016b). The first detection had to wait until the Giotto spacecraft encounter with comet 1P/Halley (1P) in 1986 (Neubauer et al., 1986). With the help of telemetry from other spacecraft’s encounters with this comet, Giotto could achieve a closest approach distance of 596 km, the closest of all 1P flybys. This also enabled Giotto to pass through the diamagnetic cavity. It was found that it was roughly spherical with a size of around 5,000 km. Solar wind ions were observed to be gradually slowed down and deflected as the spacecraft approached the comet, with some minor impact of a bow wave providing additional acceleration. A region free of solar wind ions was not observed, due to instrumental problems (Johnstone et al., 1986).

Earlier experiments with a barium release upstream of Earth in the solar wind (Valenzuela et al., 1986) had shown that the electron thermal pressure was a likely candidate to balance the magnetic pressure (Haerendel et al., 1986). However, barium has a much higher ionization rate than water and thus the cloud of ionized gas was very dense and compact, as opposed to the coma of a comet which is dense only near the nucleus, but with particles also being ionized very far upstream in the solar wind.

Cravens (1986) and Cravens (1987) found that the size and existence of the DC at 1P/Halley could not be explained by a pressure balance between the magnetic pressure on the outside and the kinetic or thermal pressure of the comet's ionosphere on the inside. Instead, they proposed that the magnetic field would be prevented from entering the innermost region of the plasma environment by ion‐neutral collisions. A simple ionospheric model was in agreement with the measured size of the cavity at Halley as well as the magnetic field profile in the DC boundary (DCB).

With the arrival of the Rosetta spacecraft at comet 67P/Churyumov‐Gerasimenko (67P), new observations of the diamagnetic cavity showed that neither the thermal pressure of the electrons, nor the simple model of the ion‐neutral friction were sufficient to explain the extension of the diamagnetic cavity at this comet (Goetz et al., 2016a, 2016b). Instead the extension of the cavity was correlated to the electron exobase, pointing to a different mechanism being relevant for the formation of this boundary (Henri et al., 2017). Although at first unexpected, this observation is consistent with observations of cold electrons in the vicinity of the cavity (Engelhardt et al., 2018; Odelstad et al., 2018; Wattieaux et al., 2020). It is also speculated that an ambipolar electric field in the DC arises due to a gradient in electron pressure, which traps the electrons in the potential well near the nucleus and thus facilitates the cooling of the electrons via electron‐neutral collisions (Eriksson et al., 2017).

There are two fundamental differences between 1P and 67P: First, while the heavy ion gyroradius *r*_*gi*_ of the cometary ions at comet 1P was small compared to the size of the structure (*r*_*gi*_ ∼ 5,000 km), the gyroradius is larger or of the same size as the structures of interest at 67P. This means the heavy ions in the entire coma at 67P are not magnetized. Behar et al. (2017) found that this results in the creation of a so‐called solar wind ion cavity, a region devoid of solar wind ions, for sufficiently high gas production rates (about 2 × 10^27^−10^28^ molecules/s). Thus a solar wind ion is deflected and decelerated far upstream of the diamagnetic cavity, and the momentum flux is conserved by accelerated cometary ions instead of solar wind ions (Nilsson et al., 2020; Williamson et al., 2020). These heavy ions together with a mix of solar wind and cometary electrons carry the magnetic field further toward the nucleus. Thus, solar wind ions are not expected to be observed near or in the DC under the conditions observed in these studies. Second, the plasma balance in the diamagnetic cavity at 1P was skewed toward photochemical equilibrium, that is, no (radial) transport of ions occurs as ions that are created are immediately removed again by recombination. At 67P, the lower densities mean that transport becomes important as well, and ions gain a nonzero radial velocity (Beth et al., 2019; Odelstad et al., 2018).

The shape of the DC was found to be roughly ellipsoidal and its surface and shape at 67P and 1P were found to be highly variable. This is mostly due to surface instabilities, e.g., possibly Kelvin‐Helmholtz instabilities at 1P (Neubauer, 1988). Both simulations (Huang et al., 2016; Koenders et al., 2015) and observations (Goetz et al., 2016a; Henri et al., 2017) also indicate the DC is irregularly shaped with protrusions or boundary surface waves extending far into the surrounding plasma. It was also found that the diamagnetic cavity expands quickly (of the order of seconds to minutes) at 67P in response to changing plasma parameters.

In a first approximation, it was thought that the DCB is a contact discontinuity with no mass transfer across the boundary. However, at 67P it was already shown that sometimes a plasma pulse can penetrate the boundary and transfer to the unmagnetized region (Hajra et al., 2017; Masunaga et al., 2019). In addition, the radially expanding low energy plasma is thought to be able to cross the boundary (Eriksson et al., 2017; Odelstad et al., 2018).

The European Space Agency's Rosetta is the only spacecraft that has ever visited a comet for an extended amount of time. It arrived at comet 67P in August 2014 and explored the comet nucleus and its environment for over 2 years, until its demise in September 2016. This allows the study of not only one parameter set, but an entire range of parameters as well as the evolution of the plasma environment. Rosetta is a relatively slow‐moving spacecraft (a few m/s with respect to the nucleus), thus any detection of a boundary or region is related to the movement of that boundary or region and not the movement of the spacecraft. Due to the constantly changing conditions at comet 67P (e.g., Edberg, Alho, et al., 2016; Edberg, Eriksson, et al., 2016; Goetz et al., 2018), Rosetta was able to observe the DC over 700 times, with intervals in the unmagnetized region from a couple of seconds up to about 40 min (Goetz et al., 2016a, 2016b). This multitude and fast succession of observations indicate that the boundary is highly unstable and can vary on very short time scales.

With this we can, for the first time, observe the diamagnetic cavity at a comet near the end of its lifetime at low gas production rates. One unexpected observation at this stage is that protons and alpha particles are detected near the DC and even inside it. This paper aims to study these observations, explain the source of the particles and explore implications of this unexpected observation for the physics of the inner coma of 67P.

## 2 Observations

### 2.1 Instrumentation

All our observations are based on measurements taken by the Rosetta spacecraft. The spacecraft accompanied comet 67P along its orbit from 3.6AU to perihelion at 1.24AU to 3.8AU. The cometocentric distances varied significantly, from the surface up to 1,500 km. Rosetta's ability to get close to the comet was determined by its ability to navigate the dusty environment, so that larger distances were necessary during higher cometary activity. Thus, with the decline in the gas production rate after perihelion, the spacecraft was able to slowly decrease the distance to the comet again. This interplay between heliocentric and cometocentric distances makes interpretation of the data very intricate.

Rosetta is equipped with a full suite of plasma instruments, the Rosetta Plasma Consortium (Carr et al., 2007). The MAGnetometer RPC‐MAG can measure the magnetic field with a cadence of up to 20 vectors/s and an accuracy of usually about 3 nT per component. Near the diamagnetic cavity this accuracy is increased to 1 nT per component (Götz, 2019). Here, we use a resampled data set with a time resolution of 1s for the magnetic field.

The LAngmuir Probe RPC‐LAP is capable of measuring the plasma density, electron temperature, plasma velocity, AC electric field, spacecraft potential and solar EUV flux, depending on the plasma state and instrument mode. For more information see, for example, Eriksson et al. (2007) and Johansson et al. (2021).

The Mutual Impedance Probe RPC‐MIP can provide the electron density and temperature. Under certain circumstances it is also possible to distinguish between two electron populations with different temperatures (Wattieaux et al., 2020). Due to Debye length constraints, MIP cannot measure low densities. This lower threshold depends on the instrument mode and the Debye length. For more details see Trotignon et al. (2007).

The Ion Composition Analyzer RPC‐ICA measures the 3D distribution function of the ions as well as the mass of the ion (Nilsson et al., 2007). A full sweep takes 192s, where 16 azimuth values are measured simultaneously, 96 energies are stepped through in 12s and then 16 elevations follow in sequence. The field of view is 360° (azimuth) by 90° (elevation) and overlaps partially with that of IES. A high‐time resolution mode (Δ*t* = 4s) was implemented as well, but this is not used in our study as it does not cover the energy range for the solar wind ions (Stenberg Wieser et al., 2017). For this study, we use the mass separated data set that distinguishes between heavy ions and light ions (Nilsson et al., 2017). There are several products available: the count rate, the differential flux, and derived ion moments, such as velocity, density, and mean (bulk) speed. The flux is used for quantitative analysis and summed over all azimuth values. The moments are calculated according to the procedure described in Williamson et al. (2020) and Nilsson et al. (2020): each full spectrum generates one moment estimate which is then assigned to the half time of the spectrum. The mean speed is calculated via the mean energy for each ICA spectrum. It represents the mean energy of the distribution and does not give any directional information.

Ion and electron spectra from the Ion and Electron Sensor RPC‐IES are used for reference and verification. RPC‐IES is capable of measuring the 3D distribution function of the electrons and ions with a field of view of 360° by 90°. However, there is some obstruction from the spacecraft and high‐gain antenna. A full angular and energy sweep takes at least 128 s. More information can be found in Burch et al. (2007) and Clark et al. (2015).

In addition, we use neutral gas densities provided by ROSINA‐COPS (Balsiger et al., 2007) to estimate the gas production rate. We assume a simple spherical model as detailed in Haser (1957) with 1 km/s neutral gas radial velocity to derive the gas production rate from the in situ measurements. All positions and fields are given in a Cometocentric Solar EQuatorial (CSEQ) coordinate system (ESA SPICE Service, 2019), unless otherwise noted.

Determining the solar wind parameters at the comet is difficult, as the Rosetta mission did not have an upstream solar wind monitor, therefore we must rely on predictions from propagation models that use measurements at other locations in the solar system (most often Earth) and a solar wind model to estimate the solar wind conditions at 67P. The angular separation between the Earth and comet 67P in the times considered here decreased from 60° to 20°. This is relatively low, so the propagation errors are also expected to be low. Here we use the 1.5D MSWIM model (Zieger & Hansen, 2008). The OMNI data set, which provides plasma and field measurements at Earth, is also used to verify the models and infer the properties of the solar wind.

### 2.2 Data

**Figure 1:**
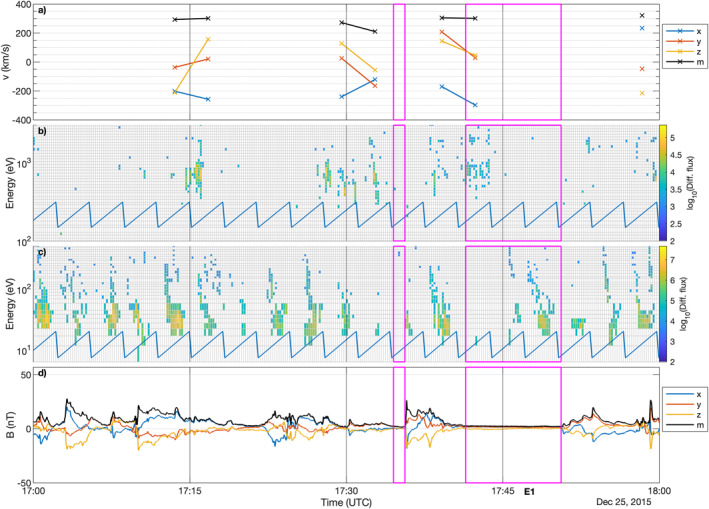
Observations of Event 1. (a) ICA proton velocity moment components and magnitude (black) in Cometocentric Solar EQuatorial (CSEQ). (b) ICA differential flux of solar wind particles. (c) ICA differential flux of heavy ions. (d) Magnetic field components and magnitude (black) in CSEQ. The magenta lines frame the times at which a diamagnetic cavity was identified. The blue line in the two middle panels is an indication of the elevation sweep of ICA, as the differential flux shown here is not summed over elevation to give higher time resolution.

**Figure 2:**
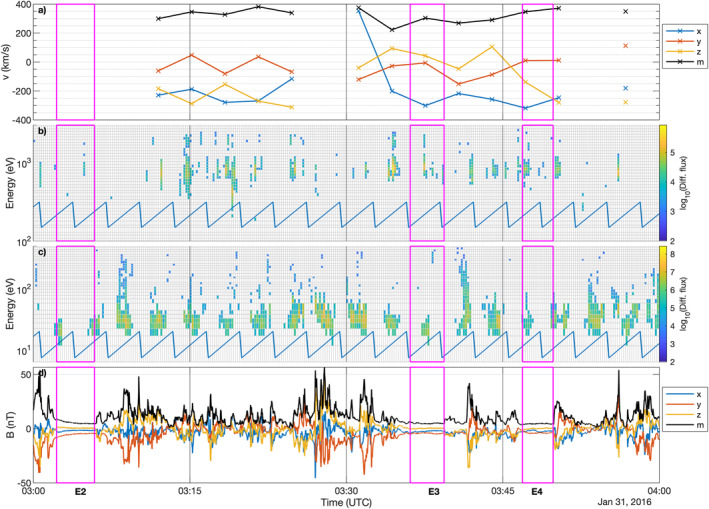
Observations of Events 2, 3, and 4. (a) ICA proton velocity moment components and magnitude (black) in Cometocentric Solar EQuatorial (CSEQ). (b) ICA differential flux of solar wind particles. (c) ICA differential flux of heavy ions. (d) Magnetic field components and magnitude (black) in CSEQ. The magenta lines frame the times at which a diamagnetic cavity was identified. The blue line in the two middle panels is an indication of the elevation sweep of ICA, as the differential flux shown here is not summed over elevation to give higher time resolution.

**Figure 3:**
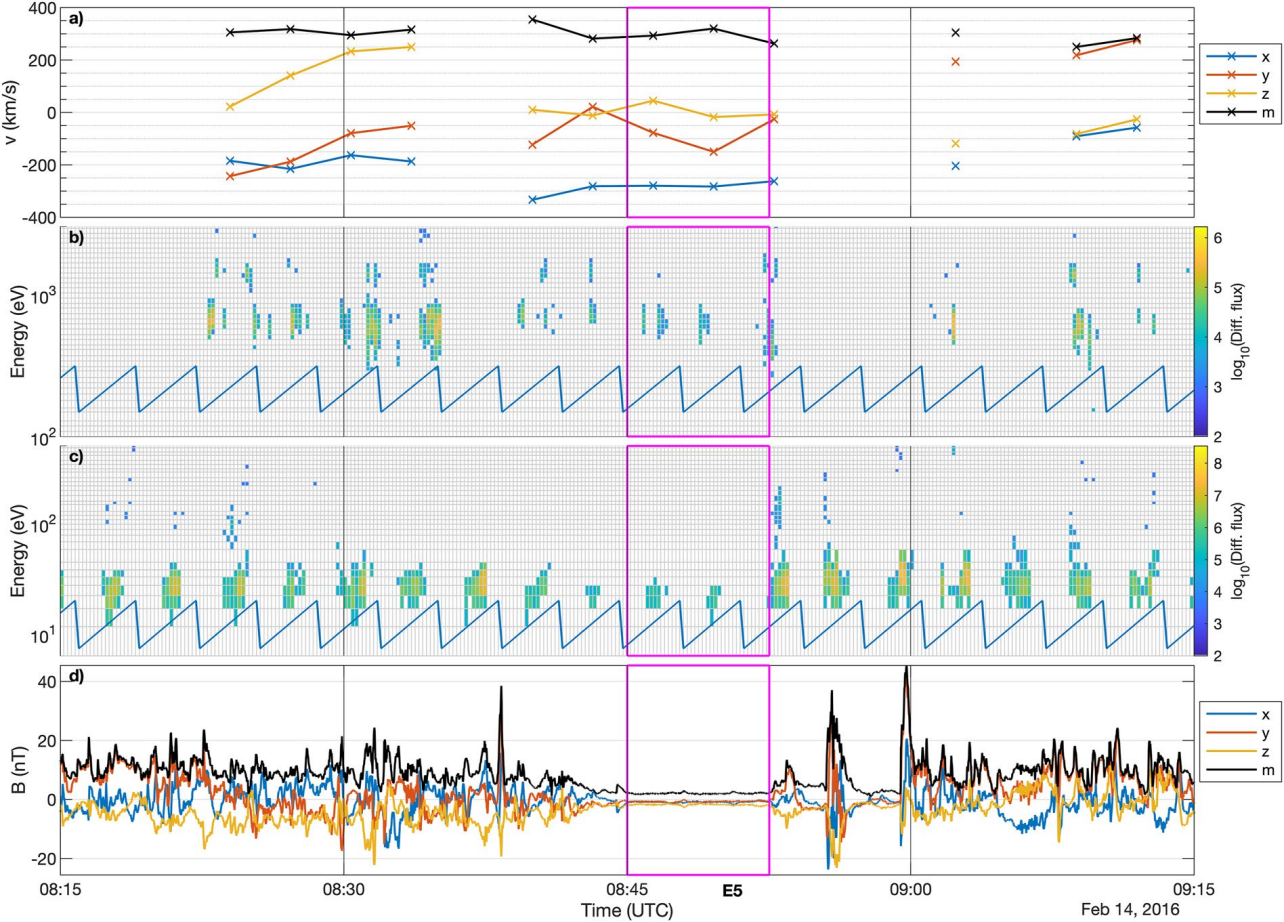
Observations of Event 5. (a) ICA proton velocity moment components and magnitude (black) in Cometocentric Solar EQuatorial (CSEQ). (b) ICA differential flux of solar wind particles. (c) ICA differential flux of heavy ions. (d) Magnetic field components and magnitude (black) in CSEQ. The magenta lines frame the times at which a diamagnetic cavity was identified. The blue line in the two middle panels is an indication of the elevation sweep of ICA, as the differential flux shown here is not summed over elevation to give higher time resolution.

Figures 1, 2, 3 show the measurements for all events, labeled E1 to E5, where protons could be detected within the diamagnetic cavity. The exact times, and key plasma and neutral gas parameters for each event are listed in Table 1. It is worth noting that this does not mean protons do not exist in the cavity at other times, because we do not have mission‐wide coverage of the solar wind ions. The magenta boxes frame the data taken inside the diamagnetic cavity, as determined using the magnetic field data. First we will discuss event E1 and then the particularities of the other events, where they differ from E1.

**Table 1:** Start and End Times of the Cavity Measurements That Also Exhibit Proton Signatures

Event	Start time *t*_*s*_ (UTC)	End time *t*_*e*_ (UTC)	*Q* (s^−1^)	*R* (AU)	*r*_*c*_ (km)	*x*, *y*, *z* (km)	*n*_*pl*_ (cm^−3^)	ICA spectra (#)	Attitude (°)
E1	25 December 2015 17:41:26	25 December 2015 17:50:35	8.4 × 10^26^	1.97	78	0.2, 76.6, 13.6	120	3	2,90,88
E2	31 January 2016 03:02:13	31 January 2016 03:05:51	2.0 × 10^27^	2.25	60	28.8, −0.9, −52.1	189	2	29,90,119
E3	31 January 2016 03:36:07	31 January 2016 03:39:22	2.2 × 10^27^	2.25	60	28.8, −0.5, −52.1	200	1	30, 90, 120
E4	31 January 2016 03:46:53	31 January 2016 03:49:49	2.3 × 10^27^	2.25	60	28.8, −0.4, −52.0	211	2	30, 90, 120
E5	14 February 2016 08:45:00	14 February 2016 08:52:31	1.3 × 10^27^	2.36	41	19.3, −31.7, 18.3	238	3	27, 90, 117

*Note.* The table also lists the average values of the gas production rate *Q*, the plasma density *n*_*pl*_, the heliocentric distance of the comet *R*, and the cometocentric distance *r*_*c*_ and positions in CSEQ *x*, *y*, *z* of Rosetta for reference. The second to last column refers to the number of ICA spectra that lie within this interval and the last column lists the attitude of the spacecraft as the angles between the three‐spacecraft axis and the Sun‐spacecraft line.

#### 2.2.1 E1: 25 December 2015

The proton observations are visible in the ICA solar wind moments (upper panel, measurement point exists inside the cavity) and in the second panel (b) in the solar wind energy spectra. Rosetta is in the diamagnetic cavity for a relatively short period and only the first full spectrum within the diamagnetic cavity shows signatures of protons in the diamagnetic cavity. However, protons are unambiguously detected in the first spectrum, and there are very small fluxes of protons also detected in the second and third spectrum. Their moments indicate that they are marginally deflected and move with speeds close to typical solar wind speeds. The energy is also typical for a marginally disturbed solar wind which has energies between ∼600 and 1,000 eV (Nilsson et al., 2015). The heavy cometary ions in the third panel exhibit no remarkable behavior, their energy is usually between 10 eV/q and a couple of hundred eV/q and no particular structure is visible. For the first spectrum within the diamagnetic cavity, there are no heavy ions observed. In the second spectrum their flux is low, while there is clear evidence of a heavy ion population in the third spectrum. The magnetic field measurements (bottom panel) show the characteristic signatures of steepened waves (Ostaszewski et al., 2020) with often sharp, large amplitude increases and gradual decreases. Two intervals were identified as diamagnetic cavity, but only one contains proton signatures.

#### 2.2.2 E2–E4: 31 January 2016

About a month after the first detection of protons in the cavity, Rosetta was able to observe protons in three successive cavity crossings. While there are signatures of protons in the energy spectra of event E2, these are quite spurious and no moments were calculated as a result. We will disregard this event from further study. For the rest of the interval, the protons behave similarly to E1, meaning they move with speeds close to typical solar wind speeds. They are mostly not deflected significantly except for one scan around 3:30 when they are almost flowing sunward (see also Scan 65 in Figure A4). The protons in the cavity are moving roughly antisunward. The cometary ion spectra show that there is a reduction in flux of the accelerated (above 40 eV) cometary ions inside and just outside (±1 spectrum) of the diamagnetic cavity. The magnetic field is again characterized by steepened waves. In the first part of the interval, the plasma density and magnetic field are highly dynamic, dominated by similar large‐scale structures as before. The only intervals of a quiet plasma are in the diamagnetic cavity.

#### 2.2.3 E5: 14 February 2016

The last event when protons could be detected in the diamagnetic cavity is also among the last events when the diamagnetic cavity could be detected at all by Rosetta (only three events occurred after that, all in the 3 days following this one but no ICA solar wind measurements were available for those later days). Proton signatures are unambiguously detected in the diamagnetic cavity. The plasma behaves very similar to the other four events. Near the diamagnetic cavity, there are large‐scale variations of the magnetic field and the solar wind ions move into a more antisunward direction. The cometary ion spectra show again that there is a reduction in flux of the accelerated (above 40 eV) cometary ions inside and just outside of the diamagnetic cavity. This reduction in flux correlates with the interval of very negative proton speed in the *x* direction.

For E1 to E5, the magnetic field signature is dominated by the characteristic steepened waves that occur near the diamagnetic cavity. However, as detailed in Appendix A, the plasma at this stage of the comet's activity also shows a magnetically quieter region, with piled‐up field, but only small amplitude variations. This is more typical of the intermediate cometary plasma environment (Goetz et al., 2022). In addition to this, the protons observed at those times are much more deflected and decelerated than those observed inside and just outside of the diamagnetic cavity.

#### 2.2.4 Control Event: 21 December 2015

In order to better understand the behavior of the protons inside the diamagnetic cavity, one may look at a control event where there are protons outside of the diamagnetic cavity but not inside of it. Figure A7 shows such an event, just 4 days before E1. This particular time was chosen because the plasma conditions are reasonably similar to those of E1–E5 and ICA is in a mode that allows for the measurement of solar wind energies. Generally, the measurements are quite similar to those discussed above. The proton flux is quite low, but sometimes still measurable. The measured protons are quite deflected and not antisunward, but still at velocities around the expected solar wind velocity. The cometary ions, electrons and density behave similarly to the other events. The magnetic field and its direction clearly show the presence of a diamagnetic cavity, and steepened wave structures outside of it. There is no “quiet” magnetic field region, without steepened waves. Therefore, the most marked difference in this event is that the solar wind protons are deflected all the time, they do not penetrate the diamagnetic cavity, and the magnetic field shows the typical steepened wave structure at all times.

#### 2.2.5 Additional Data

For all events, the influence on the particle measurements of spacecraft attitude changes was investigated, but no influence of the spacecraft pointing could be found. We therefore conclude that the proton signatures are of natural origin.

**Figure 4:**
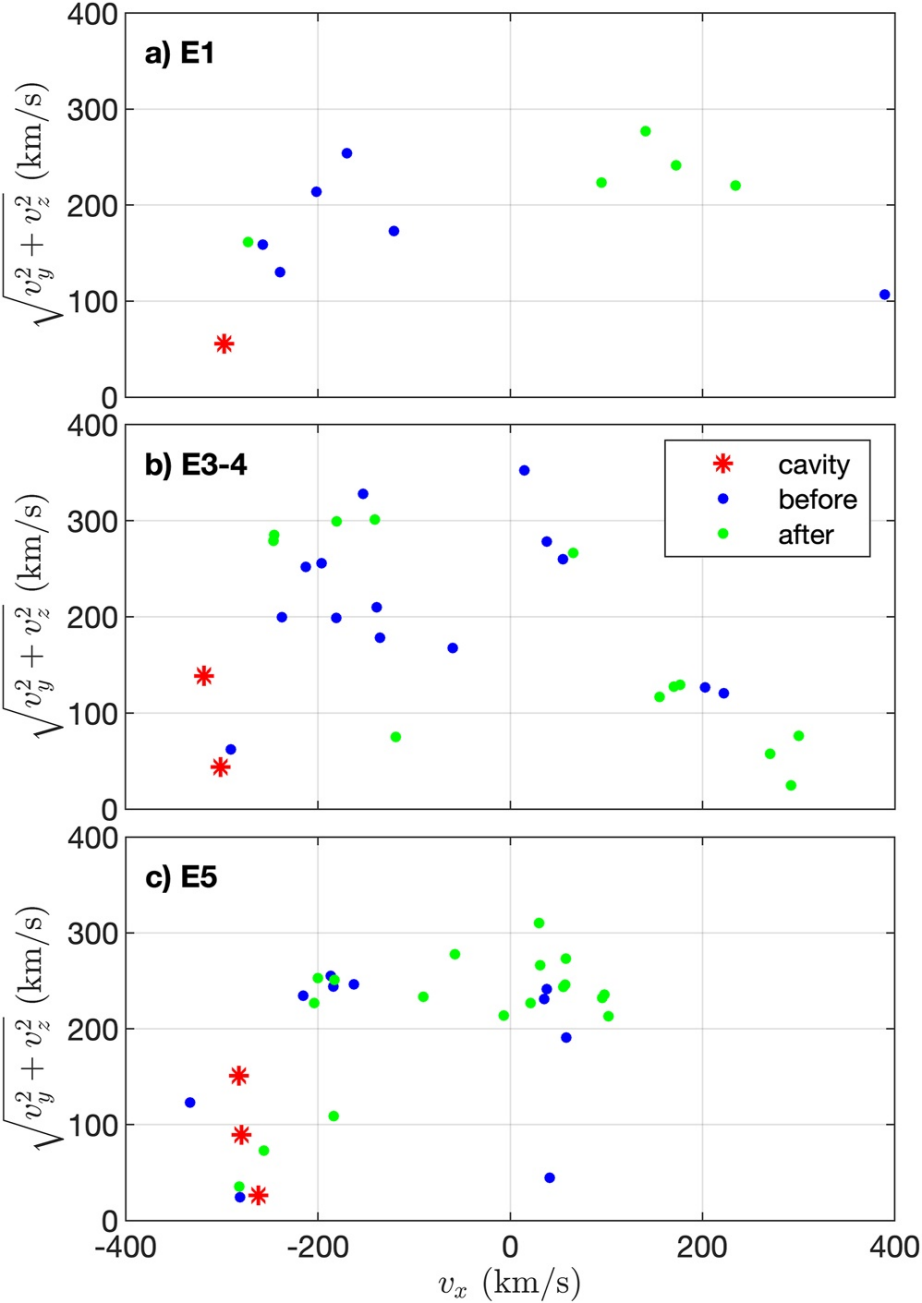
Solar wind velocity as calculated from ICA observations. The coordinate system is Cometocentric Solar EQuatorial (CSEQ), thus a high negative value of v_*x*_ indicates an antisunward moving solar wind. The value of the *y* axis indicates the deflection of the solar wind. The red stars mark the values observed in the cavity, and the blue (and green) points mark the values in the 2 h before (after) the cavity. Since E3 and E4 are closely spaced together, they are combined in a single panel.

In order to ascertain that these protons are indeed of solar wind origin and not produced locally through chemistry, we turn to the directional information that ICA provides. Figure 4 shows the ICA derived solar wind velocity in a cylindrical coordinate system. The values of the solar wind velocity that were measured inside the cavity are indicated in red and the blue and green points provide context from the 2 hr surrounding the cavity crossing. Although the solar wind protons in the entire interval in question flow in a wide variety of directions as indicated by the blue and green points, it is noticeable that the values inside the cavity are all at very negative v_*x*_ values and at low values of $\sqrt{v_{y}^{2}+v_{z}^{2}}$. This indicates that the solar wind inside the cavity is faster and more like the undisturbed solar wind than it is for the rest of the interval. Upon further examination, we also find that those values outside of the cavity that are also at very negative v_*x*_ and low $\sqrt{v_{y}^{2}{+v}_{z}^{2}}$ are all taken within a couple of minutes of the cavity crossings. Thus, the protons large velocity and directionality both indicate that the protons originate from the solar wind.

While so far, the observations shown here have focused on the protons, the solar wind He^2+^ signal, when detected, behaves very similarly, the only difference being that the fluxes are lower and thus He^2+^ ions are not detected every time protons are detected.

**Figure 5:**
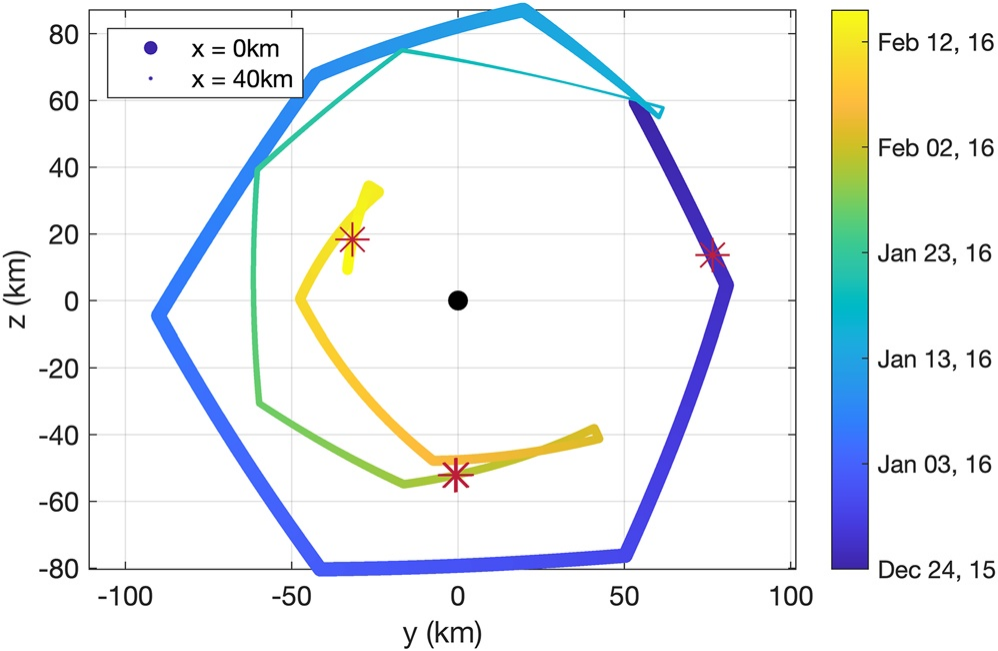
Trajectory (in Cometocentric Solar EQuatorial (CSEQ)) of the spacecraft in the *y*–*z* plane, going from blue to yellow with increasing time. The interval shown is from 24 December 2015 to 15 February 2016. The comet is indicated by a black dot. The dot size indicates the position in *x*, with smaller dots representing positions further from the comet, toward the Sun. The red stars show where protons were detected in the diamagnetic cavity along the trajectory.

The trajectory of Rosetta for the intervals in question is shown in Figure 5. Most of the time Rosetta is orbiting in a terminator orbit (*x* = 0 km), with only marginal excursions toward the dayside. This is typical for Rosetta trajectories. The events occur on all sides of the comet, there is no specific direction that is favored, but the later the event, the smaller the cometocentric distance (see Table 1). The gas production rate is similar for all events, from 0.8 to 2.3 × 10^−27^ s^−1^. These are typical values for this stage of cometary activity.

**Figure 6:**
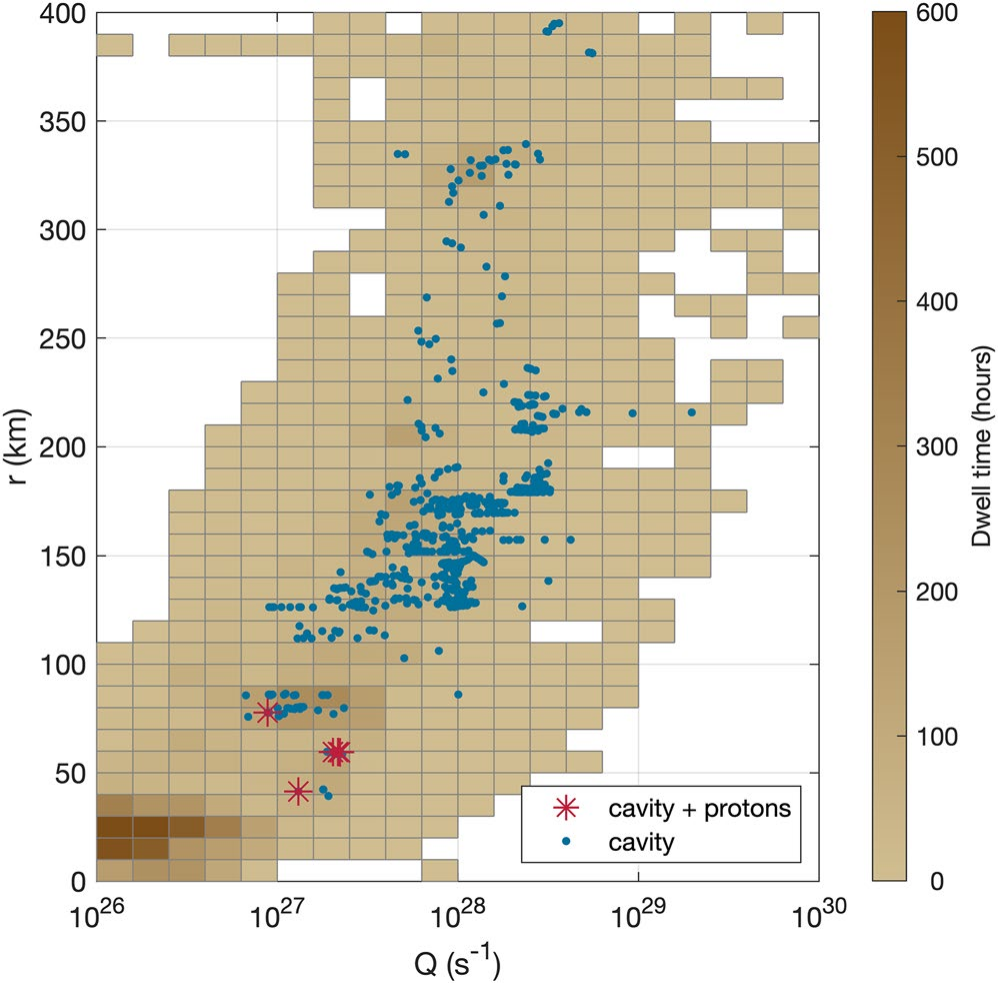
Gas production rate against cometocentric distance. The color gives the dwell time of the spacecraft during the entire comet phase of the Rosetta mission, the blue points indicate cavity events, and the red cross shows the cavity events where protons were detected.

For context, Figure 6 shows the spacecraft cometocentric distance over the gas production rate with the dwell time of the spacecraft color coded. All cavity detections are indicated and the five events where protons have been detected are marked by red asterisks. It is notable that those five events occur only in the low *Q* − low *r* regime, even though a large number of distances and production rates are covered.

## 3 Discussion

The observations presented above are surprising, as it was not expected to see any particles of solar wind origin in the diamagnetic cavity. We therefore aim to explain why this could happen, under which conditions and what the implications are for the physics of the cometary plasma.

### 3.1 Crossing the Diamagnetic Cavity Boundary

We can calculate the gyroradius of the protons for typical magnetic field strengths and solar wind ion velocities, with *B* = 10 nT and v = 300 (50) km/s, then the gyroradius *r*_*g*_ is approximately 300 (50) km. Considering that Rosetta is less than 80 km from the nucleus for all events, it stands to reason that the DCB has a width of at the most a few tens of kms. In fact, observations (Neubauer, 1988) and simulations (Koenders et al., 2015; Rubin et al., 2012) found boundary thicknesses of around 25 km for cavity extensions larger than what is observed here. Thus, the gyroradius of the protons is much larger than the boundary and the ions should easily traverse it if they are present in the inner coma, i.e., the region just upstream of the diamagnetic cavity. Cometary ions from outside of the diamagnetic cavity are known to be able to move through the boundary unimpeded (Hajra et al., 2018; Masunaga et al., 2019).

### 3.2 Protons in the Inner Coma

We have seen that if the protons from the solar wind can reach the inner coma, they can easily traverse into the cavity. The question remains how the protons can reach the inner coma in the first place and not be significantly deflected nor decelerated.

In addition, we need to be able to explain why the cavity is observable. As Rosetta is almost stationary with respect to the comet at the time of our events, the diamagnetic cavity needs to move over the spacecraft to be detected. According to Goetz et al. (2016a), this happens either if there is a genuine expansion of the entire cavity or if the boundary shape is highly variable, that is, there is an oscillating boundary, resulting in many crossings in a short time period. While E1 and E5 are isolated events that can only be caused by an expansion, E2–E4 all occur within 1 hr. In an interval on 20 November 2015 taken from Goetz et al. (2016a), 25 events were detected in the span of 6 hr, indicating a wavy cavity boundary with a local bulge passing over the spacecraft repeatedly. The average period is 1,400 s with a standard deviation of 500s. The period of E2–E4, 2700 ± 700 s, is almost twice as long and does not agree with the period of 20 November within 1σ. We therefore assume that these events are also due to a genuine cavity expansion.

Thus, in addition to the first condition of minimal deflection, a second condition needs to be satisfied for all events: whatever pushes the protons inward also needs to allow the diamagnetic cavity to expand. Otherwise, Rosetta would not be able to observe protons during these diamagnetic cavity events. In the following discussion, we will refer to this as condition A (solar wind ions not deflected/slowed down) and condition B (cavity expands).

Below, we attempt to explain why the protons can reach the inner coma while the diamagnetic cavity expands.

#### 3.2.1 Outburst

Since we derive the gas production rate *Q* from in situ measurements of the neutral density, an outburst would increase *n*_*n*_ and also *Q* (Figure 6). This is not seen in these intervals. We therefore rule out a neutral gas outburst as the trigger for the cavity expansion.

#### 3.2.2 ICME/CIR

Edberg, Alho, et al. (2016) showed that the arrival of an Interplanetary Coronal Mass Ejection (ICME) can compress the plasma environment of the comet and subsequently allow the solar wind to get closer to the nucleus than usual. Thus, we investigate the possibility that an ICME or a Corotating Interaction Region (CIR), another solar wind structure with high dynamic pressure, impacted the comet at the times in question. The structure of an ICME often includes a high density (and therefore high dynamic pressure) region, followed by a shock and a slow solar wind region (Tsurutani et al., 1988), as observed from a stationary object in the solar wind. CIRs behave similarly, but in this case the cause of the high dynamic pressure is a higher than usual solar wind velocity. CIRs are often bound by forward and reverse shocks on either side (Smith & Wolfe, 1976). Both ICMEs and CIRs are often associated with increases in the magnetic field strength. ICMEs are often also associated with Forbush Decreases (FDs), short lived (a few hours) decreases in the energetic particle environment (Cane, 2000).

**Figure 7:**
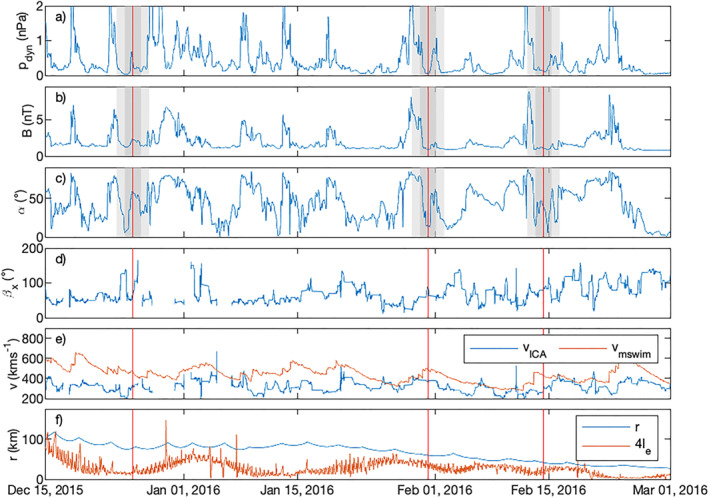
Output of the MSWIM solar wind model (top three panels) and Rosetta data (bottom three panels). From top to bottom: (a) solar wind dynamic pressure, (b) magnetic field magnitude, (c) angle *α* between the magnetic field and the solar wind direction, (d) angle *β*_*x*_ between the Cometocentric Solar EQuatorial (CSEQ) *x* axis (antisolar wind direction) and the ICA proton velocity, (e) ICA proton mean speed and MSWIM predicted solar wind speed, (f) cometocentric distance of the spacecraft and electron collision scale *l*_*e*_ multiplied by a factor 4, for visibility. The three red vertical lines indicate the timing of the five events. The areas shaded in lighter and darker gray, represent 1 and 2 days of model uncertainty around the events, respectively.

The observations of the solar wind velocity during events E1–E5 show no significant enhancement in the overall solar wind velocity or density. The Standard Radiation Monitor (SREM) on Rosetta also did not detect an FD. The magnetic field strength is also within normal values during the time shown here. Now, it should be noted that these measurements were taken deep within the coma of 67P, and the interaction of the solar wind with the cometary plasma will have changed the solar wind. However, Edberg, Alho et al. (2016), Edberg, Eriksson, et al. (2016), Hajra et al. (2018), and Goetz et al. (2018) all showed that some signature of a CIR or ICME always remains visible even close to the comet for a few hours at least. Thus, it is unlikely that an ICME or CIR impacted 67P in the interval in question. The first panel of Figure 7 shows the dynamic pressure of the solar wind at comet 67P as predicted by the MSWIM model. Considering that solar wind propagation models usually have uncertainties of the order of tens of hours, a direct association with the events in question should be made with caution. While enhancements of the dynamic pressure are predicted in the interval in question, they are just outside the maximum model uncertainty of 2 days around the events (shaded in light gray in Figure 7) and are therefore unlikely to be a contributing factor. When combined with the result of the in situ measurements, we can conclude that it is unlikely to be of consequence for this investigation.

Another sign that this scenario is unlikely comes from the fact that the cavity is detectable at all. Figure 6 clearly shows that the diamagnetic cavity at 67P at the time investigated here (December 2015 to February 2016) must be very small already, due to the low outgassing rates. As an ICME and CIR would presumably compress the diamagnetic cavity similarly to the solar wind ion cavity, we would not be able to observe it at all.

So, while an ICME/CIR impact could explain condition A, it cannot explain condition B, and there is no clear sign that such a structure came near the comet at the times in question. We therefore discard this theory.

#### 3.2.3 Charge Transfer

One possible mechanism that could allow the ions to reach the diamagnetic cavity without being deflected (condition A) is that of charge transfer, a process that has been observed at Mars (Halekas et al., 2015). A solar wind proton upstream of any cometary plasma boundary could be neutralized (electron capture), thus penetrating unimpeded through any plasma boundaries, and then ionized again (electron stripping) deeper in the cometosphere while maintaining its initial speed and direction: H^+^ + *e* ^−^ → H → H^+^ + *e* ^−^.

To verify or exclude this mechanism, we calculate the likelihood of the solar wind protons undergoing both of these processes. We use a simple model based on the particle continuity equation presented in Simon Wedlund et al. (2016) with cross sections *σ*_*CX*_ taken from Simon Wedlund et al. (2019). To cover a range of solar wind velocities, cross sections for particle impact speeds between 200 km/s and 1,000 km/s are included. The ratio of the ion flux lost due to charge‐exchange $F_{X^{n+}}\left(\bar{r}\right)$ to the initial solar wind flux $F_{X}^{SW}$ is:

(1) $$R_{X}^{SW}\left(\bar{r}\right)=\frac{F_{X^{n+}}\left(\bar{r}\right)}{F_{X}^{SW}}=e^{-\sigma_{CX}\;I_{n}\left(\bar{r}\right)}$$

The column density of neutrals along the solar wind ion trajectory is defined as a line of sight integration:

(2) $$I_{n}\left(\bar{r}\right)=\int_{\bar{r}}^{\infty }n_{n}\left(r\right)\;dr$$

The neutral gas density *n*_*n*_ is described by a simple spherically symmetric model (Haser, 1957):

(3) $$n_{n}\left(r\right)=\frac{Q}{4\pi r^{2}u_{n}}$$

where *u*_*n*_ = 1,000 m/s is the neutral gas speed, *Q* is the gas production rate, and *r* is the cometocentric distance.

Using the cross section for electron capture (H^+^ → H) *σ*_10_ in Equation 1 gives the number of protons that have been converted to neutral atoms. From this number, the next step is to apply the model again, with cross sections *σ*_01_ for the second reaction, the stripping of an electron (H → H^+^). Thus, the ratio of protons that have undergone both reactions to the initial flux of solar wind protons is:

(4) $$R_{X}^{SW}\left(\bar{r}\right)=e^{-\sigma_{01}\;I_{n}\left(\bar{r}\right)}e^{-\sigma_{10}\;I_{n}\left(\bar{r}\right)}.$$

This model is applied to all five events with the gas production rates as given in Table 1. The values for the collisional cross sections are dependent on the initial solar wind velocity; they can be found in Table 2 for a wide variety of solar wind velocities. The integration was run from a distance from the comet *r* of 10–10000 km. For this approximation, only charge transfer from H^+^ to neutral H and back to *H* ^+^ was considered. Leaving out the potential for repeated electron capturing to form H^−^ (Burch et al., 2015), this model thus overestimates the remaining solar wind proton flux.

**Table 2:** Collision Cross Sections for the Electron Capture (*σ*_10_) and the Electron Stripping (*σ*_01_) Reactions for Protons at Different Impactor Speeds v

v (km/s)	*σ*_10_ (m^2^)	*σ*_01_ (m^2^)
200	2.24 × 10^−19^	3.80 × 10^−22^
400	1.79 × 10^−19^	2.64 × 10^−23^
600	1.46 × 10^−19^	6.97 × 10^−22^
800	1.30 × 10^−19^	1.24 × 10^−22^
1,000	1.21 × 10^−19^	1.82 × 10^−22^

**Figure 8:**
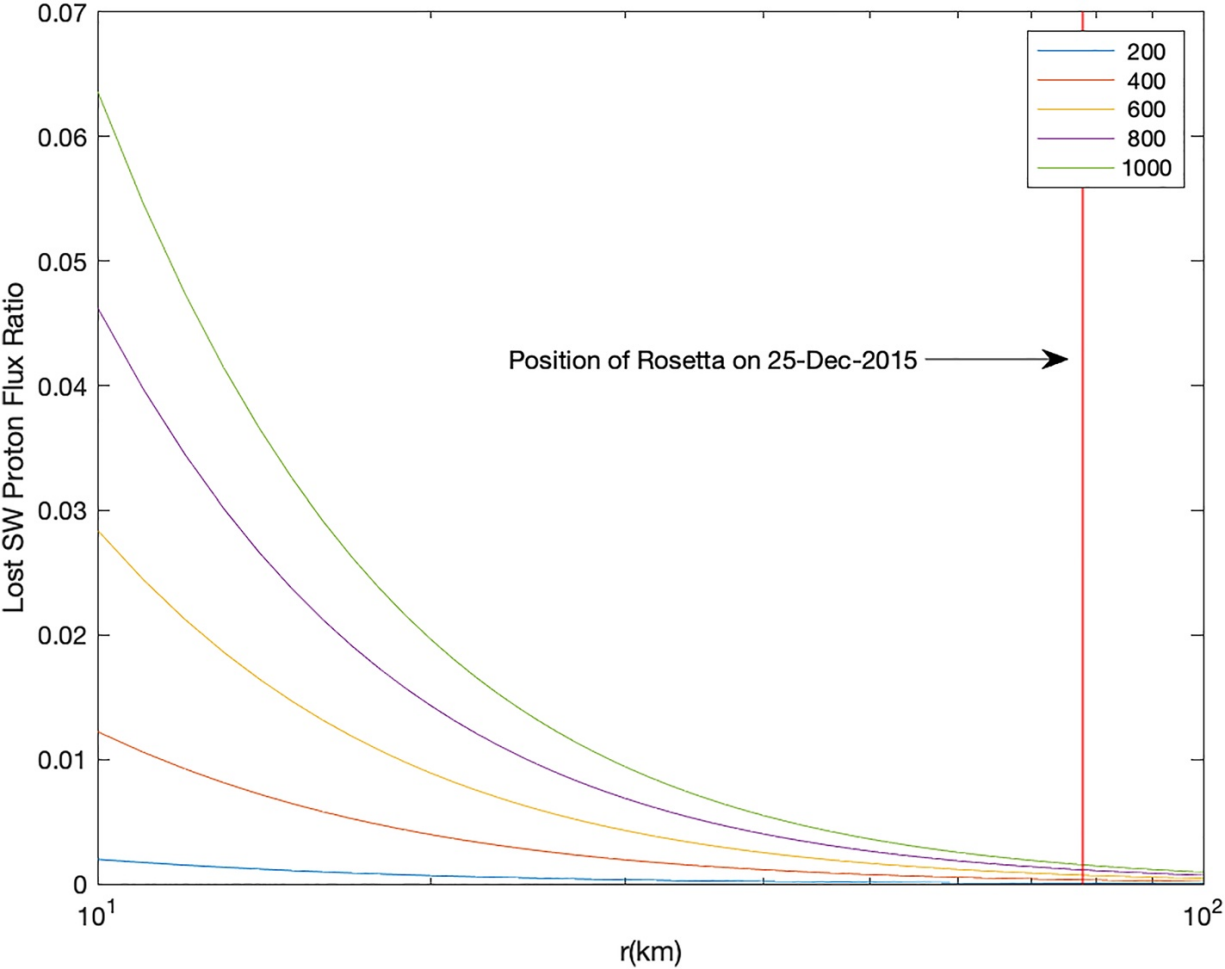
Flux of protons that have undergone electron capture and subsequently electron stripping, normalized by the upstream proton flux. The flux depends on the impactor speed (=solar wind speed), therefore a range of parameters were used (see legend). At Rosetta's position (red vertical line), the flux never reaches above 2% of the incident solar wind flux. The model uses the gas production rate given for event E1.

Figure 8 shows the flux of the protons undergoing two charge transfers, which at Rosetta's location (red) is 2% (0.02) or less of the original flux for the plasma parameters at event E1. The parameters were very similar for E2‐E5 and the proton flux never reaches significant levels (see Appendix Figures A8 and A9).

It is important to note that this scenario assumes that charge fractions are at equilibrium. However, the dynamic nature of the plasma around 67P may complicate matters and a more dynamic approach may be necessary. The collision probability for a neutral hydrogen atom to charge transfer back to a proton can be evaluated as

(5) $$P∼\sigma_{01}\;n_{n}\left(r\right){\;v}_{\text{sw}}\;\Delta t,$$

where v_sw_ is the original proton/hydrogen bulk speed and Δ*t* is the interaction time (likely short because of the small scale—10s of km—of the cavity). This probability is very small, of the order of 10^−3^Δ*t* for a typical neutral density *n*_*n*_ = 10^14^ m^−3^, v_sw_ ∼ 400 km/s and the cross section *σ*_01_ as given in Table 2. Consequently, the equilibrium solution presented above likely constitutes an upper estimate.

A quantitative investigation of these scenarios, including photoionization and electron impact ionization as a supplementary way to create protons in the DC from hydrogen crossing the DC boundary, is left for future study, because it requires the use of self‐consistent particle models such as hybrid simulations or a test particle simulation with realistic fields and boundaries. Even with this limited equilibrium calculation, we can conclude that repeated charge transfer reactions are most likely not the source of the protons appearing in the diamagnetic cavity.

#### 3.2.4 Parallel IMF and Solar Wind Velocity

What other mechanisms could cause the protons to be less deflected and slowed down? Deflection of the protons is related to the solar wind convective electric field and the pick‐up of cometary ions. Usually, the newborn cometary ions, initially at rest with respect to the background plasma, far upstream of the comet are accelerated by the convective electric field. If the gyroradius of the cometary ions is large, this leads to all cometary ions traveling in the same direction. In order to conserve momentum, the solar wind ions are deflected in the opposite direction. This pick‐up mechanism has been observed at 67P (Behar et al., 2016) and reproduced in simulations (Alho et al., 2019; Deca et al., 2017; Koenders et al., 2016). This way of cometary ion pick‐up relies on there being a convective electric field ${\vec{E}}_{c}$, and since the field is determined by

(6) $${\vec{E}}_{c}=-{\vec{v}}_{\text{sw}}\times {\vec{B}}_{\text{IMF}}$$

there is an obvious case where $\vec{E_{c}}$ is zero: that of the interplanetary magnetic field ${\vec{B}}_{\text{IMF}}$ being parallel to the solar wind velocity ${\vec{v}}_{\text{sw}}$. In this case, mass‐loading is achieved by wave‐particle interaction, where the waves are caused by a beam‐like particle distribution function (Goldstein et al., 1990). Then there is no preferred direction for the particles and therefore no deflection should be observable.

Although many studies exist on the kind of instability and resonances associated with the ${\vec{v}}_{\text{sw}}\|{\vec{B}}_{\text{IMF}}$ case, not much is known about the influence of such a configuration on the large‐scale structures in a comet's environment. Nevertheless, some pointers can be found in the literature.

We first assess condition A: no solar wind deflection. The absence (or almost absence) of the convective electric field means that other mechanisms are responsible for mass‐loading, the question is if these mechanisms are as efficient as that of convective field pick‐up. Koenders et al. (2013) performed hybrid simulations of the cometary environment with a variety of solar wind parameters, among which was the angle between the solar wind velocity and magnetic field (*α*). As this study was focused on determining the stand‐off distance of the bow shock at 67P, no other boundaries were investigated. However, it still allows to assess the efficiency of mass‐loading by using the stand‐off distance as a proxy: if the bow shock is close to the comet, mass‐loading is inefficient because it takes the solar wind flow a longer time to reach the critical point at which a shock is formed. Koenders et al. (2013) find that the bow shock stand‐off distance decreases with decreasing angle *α*. That indicates that, as the interplanetary magnetic field becomes more and more parallel to the solar wind velocity, mass‐loading becomes less efficient. This in turn means that the deflection and deceleration of the solar wind is less pronounced or even absent. Consequently, ${\vec{v}}_{\text{sw}}\|{\vec{B}}_{\text{IMF}}$ is compatible with condition A. While cometary plasmas are unique in the solar system, the Martian plasma environment can be very similar. Fowler et al. (2022) found that under conditions where they speculate that the IMF is aligned with the solar wind velocity, undeflected and marginally decelerated protons can be found in the ionosphere of Mars. This is in agreement with our hypothesis for the cometary environment.

Second, we assess condition B: expansion of the diamagnetic cavity. Although the mechanism creating the diamagnetic cavity is not known in detail, Goetz et al. (2016b) and Götz (2019) found that the diamagnetic cavity size depends on the local gas production rate and weakly on the average magnetic field just outside of the diamagnetic cavity. Since the possibility of an outburst was already ruled out as a cause for the expansion of the diamagnetic cavity, only the dependence on the magnetic field strength remains to be investigated.

A simple picture of the diamagnetic cavity boundary is the following: The incoming magnetic field is tied to the solar wind electrons and the pick‐up ions, which are slowed down until the flow reaches a stagnation point. This causes a magnetic pressure gradient, which is balanced by either ion‐neutral friction, electron‐neutral friction or plasma pressure or another as of yet unknown process. Thus, the magnetic pressure is a major contributor to what controls the size of the diamagnetic cavity.

Gombosi et al. (1994) performed MHD simulations on an axisymmetric (with respect to the solar wind velocity) grid, with solar wind conditions where $\left.{\vec{v}}_{\text{sw}}\right\|{\vec{B}}_{\text{IMF}}$. While they did not investigate specifically the influence of the angle between the magnetic field and solar wind velocity, we can still make inferences by comparing their results with observations and other simulations where *α* ≫ 0°. Since the simulation uses parameters that are appropriate for a Halley‐type comet, we use the observations at comet Halley to compare to. The main difference in the magnetic field profile as presented in Gombosi et al. (1994) and the profile reported by Neubauer (1987) is the lack of a pile‐up region in front of the diamagnetic cavity. While the observations (for *α* ≫ 0°) show an increase in the magnetic field just before the diamagnetic cavity, this increase is missing in the simulations (Figure 4 in Gombosi et al., 1994). The solar wind flow slows down as it approaches the diamagnetic cavity and the magnetic field is aligned with the flow. Since the magnetic field is divergence‐free, it cannot be compressed along the field line: *∂*_*x*_ *B*_*x*_ = 0. Thus, at the sub solar point there cannot be any pile‐up of the field. This obviously changes when considering the flow of the solar wind away from the Sun‐comet line, but the pile‐up there is also much smaller than it would be in the *α* ≫ 0° case (see Figure 6 in Gombosi et al., 1994). We infer that for ${\vec{v}}_{\text{sw}}\|{\vec{B}}_{\text{IMF}}$ the magnetic field strength in the pile‐up region is smaller than for the classical case, which in turn reduces the magnetic pressure, which then allows the diamagnetic cavity to expand. Thus, condition B is satisfied. We would also like to point out that Chang et al. (2020) came to similar conclusions when observing the impact of ${\vec{v}}_{\text{sw}}\|{\vec{B}}_{\text{IMF}}$ on the magnetization of the venusian ionosphere. They observed several cases where the ionosphere became demagnetized due to the weakening of the magnetic field pile‐up, very similar to what was observed in the simulations above and what we believe is the situation during the events presented here.

Consequently, it seems that a parallel magnetic field in the solar wind could explain the unusual proton signatures in the diamagnetic cavity. It is also worth noting that a consequence of the reduced mass‐loading efficiency in the parallel field configuration would also be a lack of accelerated cometary ions. This fits the observations at least for event E2, E4, and E5, adding evidence in support of our hypothesis.

**Figure A3:**
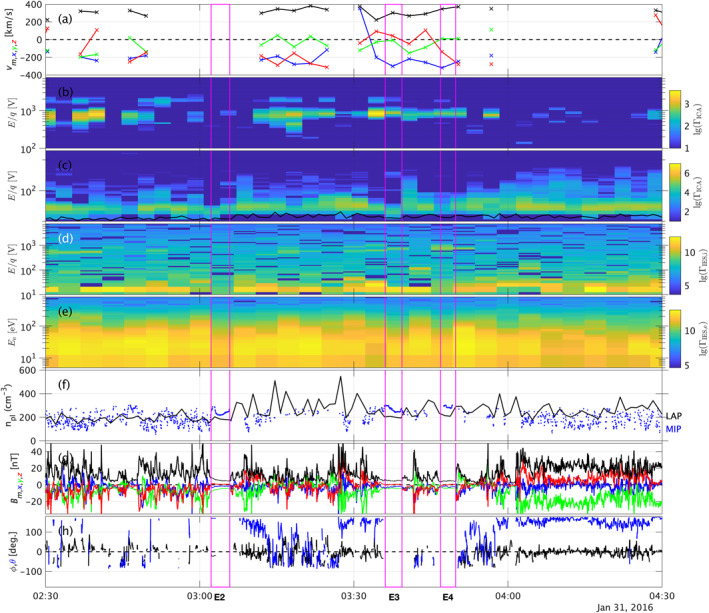
Observations for E2, E3, and E4. From top to bottom: (a) solar wind moments, (b) solar wind energy spectra (ICA differential flux averaged over all angles, cm^(−2)^s^(−1)^sr^(−1)^), (c) heavy ion energy spectra (ICA, same units as (b)) and the negative of the spacecraft potential as a black line, (d) ion energy spectra (IES), (e) electron energy spectra and the summed flux as a white line, (f) plasma density from LAP and MIP, (g) magnetic field, and (h) magnetic field cone and clock angle. The magenta lines indicate the interval when Rosetta was in the diamagnetic cavity.

**Figure A4:**
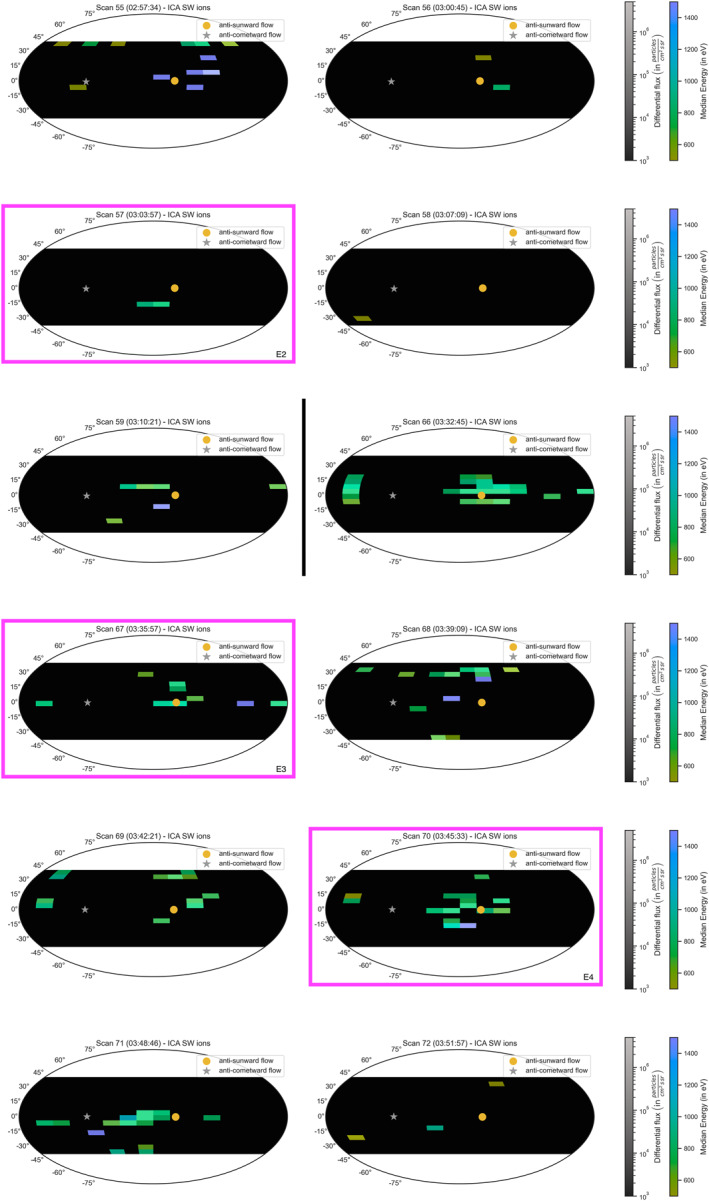
Elevation‐azimuth resolved ICA solar wind spectra for E2, E3, and E4. The color gives the mean energy in each bin and the brightness indicates the differential flux. The magenta boxes indicate which spectra are associated with the diamagnetic cavity. The coordinate system is that of the ICA field of view. A black line denotes a larger gap in time between event E2 and E3.

**Figure A7:**
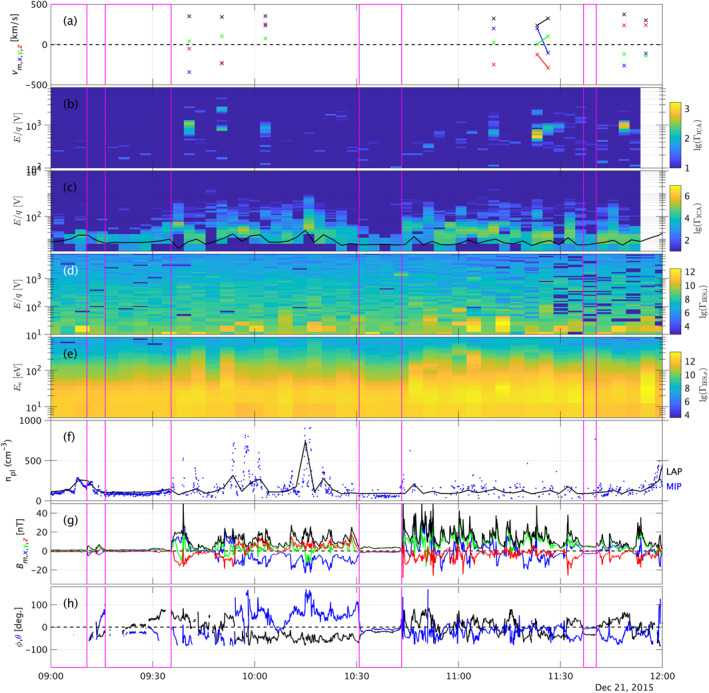
Observations of a cavity crossing without proton signatures. From top to bottom: (a) solar wind moments, (b) solar wind energy spectra (ICA counts), (c) heavy ion energy spectra (ICA counts) and the negative of the spacecraft potential as a black line, (d) ion energy spectra (IES), (e) electron energy spectra and the summed flux as a white line, (f) plasma density from LAP and MIP, (g) magnetic field, and (h) magnetic field cone and clock angle. The magenta lines indicate the interval when Rosetta was in the diamagnetic cavity.

**Figure A8:**
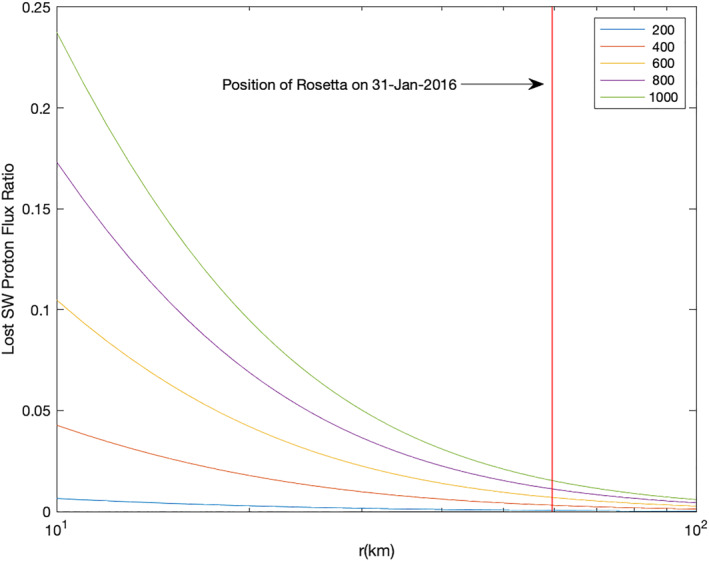
Flux of protons that have undergone electron capture and subsequently electron stripping, normalized by the upstream proton flux. The flux depends on the impactor speed (=solar wind speed), therefore a range of parameters were used (see legend). At Rosetta's position (red vertical line), the flux never reaches above 2% of the incident solar wind flux. The model uses the gas production rate given for event E2–E4.

**Figure A9:**
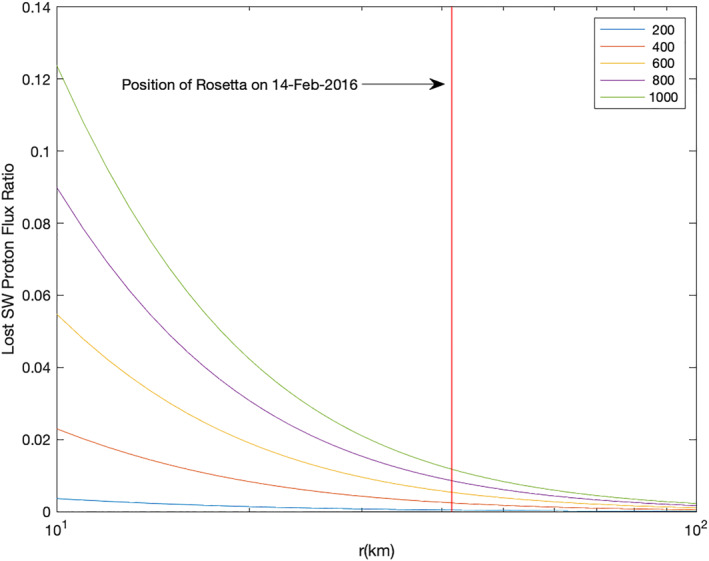
Flux of protons that have undergone electron capture and subsequently electron stripping, normalized by the upstream proton flux. The flux depends on the impactor speed (=solar wind speed), therefore a range of parameters were used (see legend). At Rosetta's position (red vertical line), the flux never reaches above 2% of the incident solar wind flux. The model uses the gas production rate given for event E5.

The question remains why there are protons outside the cavity, but not inside, for the control event. Theoretically, the gyroradius of these protons should also be larger than the boundary width and therefore they should penetrate it. In order to better understand the situation, we have used a simple toy model as described in Appendix A3 to calculate the ion trajectories near the diamagnetic cavity. We note that for the control event, the velocity of the protons is smaller than for E1–E5. We therefore model the motion of fast (80 km/s) and slow (8 km/s) protons near the cavity boundary. The exact values of the velocities in the model are of no consequence for the results, as this is merely an illustration of the two different scenarios that is relatively robust against changes in the input parameters. As shown in Figure A3, protons can penetrate the cavity in both cases, if there is no other influence on the particles, that is, no electric field. However, we know from previous findings by, for example, Vigren et al. (2017) and Odelstad et al. (2018) that there is a radially outward directed ambipolar electric field in the cavity and the region just outside of it. If we include such an electric field in the model, it becomes clear that the slower ions are expelled from the cavity, because their energy is insufficient to overcome the potential barrier of the electric field. The fast ions are decelerated, but to a smaller degree and are therefore still detectable in the diamagnetic cavity. If we attribute the more effective deceleration of the protons in the control event to the presence of a convective electric field, this implies that the protons in that case cannot cross the boundary and are indeed expelled from the cavity as observed. The lack of deceleration in the parallel field case makes it possible for the faster protons to cross the boundary and be observable in the diamagnetic cavity.

Although solar wind models for the comet are not as accurate as we would wish for, we will attempt to infer the angle of the magnetic field to the solar wind velocity from models. A preliminary analysis of OMNI solar wind data at Earth confirms that such a configuration is possible in the solar wind in general. Figure 7 shows the magnetic field strength (panel b) and the angle *α* as predicted by the MSWIM model. There are several short intervals (minutes to hours) when *α* is close to zero (indicating ${\vec{v}}_{\text{sw}}\|{\vec{B}}_{\text{IMF}}$). Therefore, generally, this configuration is possible at the comet for intervals larger than the proton transit time (order of tens of seconds). Specifically, for the events when protons are observed in the diamagnetic cavity (vertical red lines), there is a signature with a low *α* value within 1 day of the model. This is much closer than the enhancements in dynamic pressure that could indicate an ICME/CIR, as discussed above. Thus, there is at least some evidence that parallel IMF and solar wind velocity could be the explanation for our proton events. However, without a more accurate model, it is impossible to say with certainty that E1–E5 correspond to low α periods in the solar wind. The question is then, why are there no observations of protons in the cavity for other intervals where the model predicts low values of α? Panel f) of Figure 7 shows the cometocentric distance and the electron collision length scale *l*_*e*_ as calculated according to Henri et al. (2017). For other intervals with low α, either the electron collision length, which scales with the diamagnetic cavity size, is small or the cometocentric distance is large. It is therefore more unlikely to observe protons in the diamagnetic cavity. Our theory predicts that any time the modeled α is low, the measured solar wind should be less deflected and less decelerated. We have therefore calculated the angle *β*_*x*_ between the measured solar wind velocity and the Sun‐comet direction (as an analogue to the undisturbed solar wind velocity). *β*_*x*_ is shown in panel d) of Figure 7 and the measured mean solar wind speed is shown in blue in panel e). Contrary to our theory there is no correlation between the modeled *α* and the velocity. There is some indication that when the model predicts a low *α*, the angle *β*_*x*_ increases. But the resolution and accuracy of the model output is not sufficient to associate solar wind structures directly to our events. Behar et al. (2017) investigated the mass‐loading of the solar wind flow at the comet over time and found that while there is strong deflection with increasing cometary ion number density, the deceleration of the solar wind is not as strong. From this they concluded that momentum transfer is more efficient than energy transfer. This could explain why there is a better correlation between the two angles than there is between the mean speed (as a representation of energy) and the solar wind model angle. As an additional check, we have added the predicted solar wind speed in panel (e). Ideally, we would expect the measured speed to be consistently lower than the solar wind speed, as it is mass‐loaded and decelerated but it should still follow the general trend of the prediction. Overall the correlation between the two is poor, however, one has to take into account the time shift and uncertainties of the model. For example, in the latter half of January and first half of February, the predicted speed seems to increase when we observe increases in the observed proton speed. This indicates that the prediction seems to be roughly correct, if not accurate enough to assign individual events to each other.

## 4 Conclusions

Using data from the Rosetta Plasma Consortium we have identified five events where protons are observable inside the diamagnetic cavity. The observations indicate that:

- These protons originate in the solar wind and penetrate the inner coma, where they cross into the diamagnetic cavity.
- The observations of the diamagnetic cavity otherwise are in line with other diamagnetic cavity events.
- The protons are more antisunward or less deflected than normal for that activity level.
- These events occur at intermediate gas production rates and close to the nucleus.

We have found that ions crossing the diamagnetic cavity boundary is consistent with previous observations. Consequently, the main question to be answered is why solar wind protons can move almost unimpeded into the inner coma. We discussed several hypotheses and could successively exclude a CME or CIR impact, a neutral gas outburst, and charge transfer mechanisms as the triggers for this specific situation.

**Figure 9:**
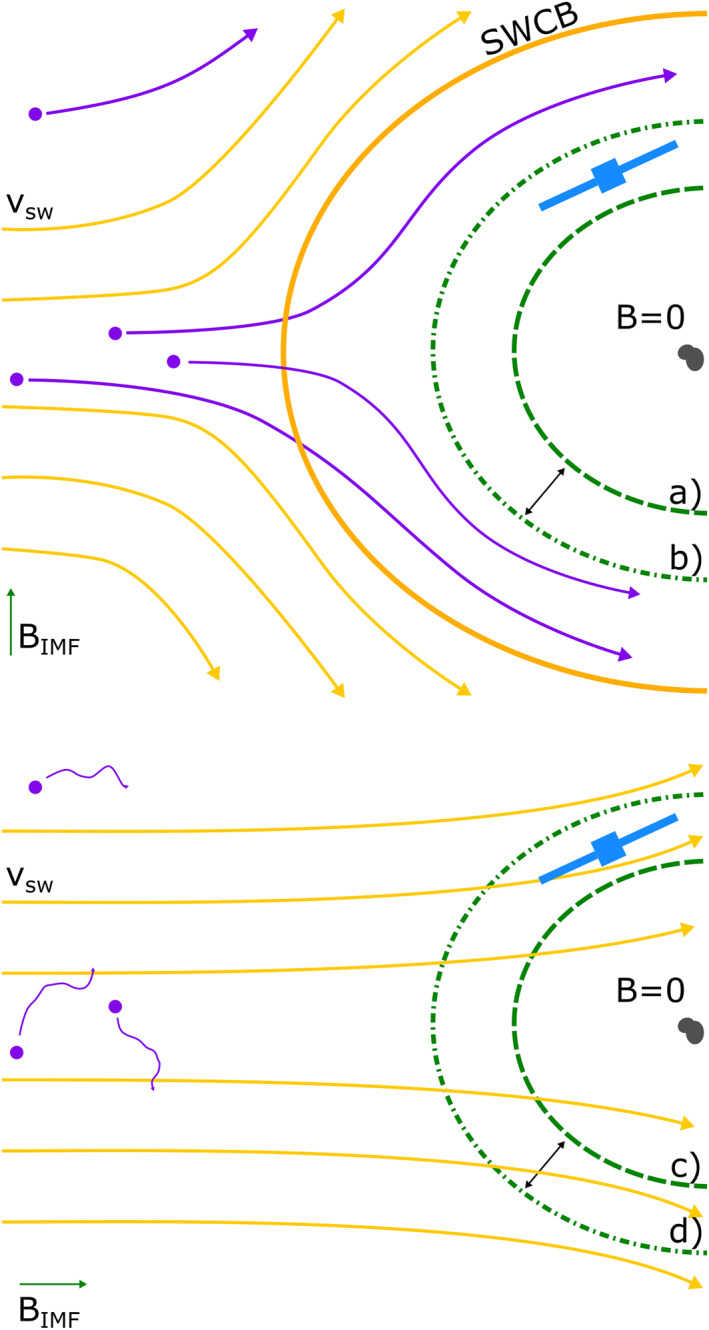
Purple particles and arrows: newborn cometary ions and their trajectories. Yellow arrows: solar wind ion flow. Orange boundary: solar wind ion cavity boundary. Green dashed and dash‐dotted line: diamagnetic cavity boundary at two different time intervals. Top: normal observations of the diamagnetic cavity, within the solar wind ion cavity. Bottom: Case with protons near and in the diamagnetic cavity.

At this moment, the most likely hypothesis is that a solar wind configuration where the solar wind velocity is parallel to the interplanetary magnetic field leads to the protons not being deflected and slowed down, enabling them to reach the inner coma. Figure 9 is a sketch of the “normal situation,” where there is a nonzero angle between $\vec{v_{\text{sw}}}$ and $\vec{B_{\text{IMF}}}$ (upper panel) and the $\vec{v_{\text{sw}}}\|\vec{B_{\text{IMF}}}$ situation (lower panel). Normally, the solar wind ions are deflected as the newly created cometary ions are picked up by the convective electric field. Therefore, the heavy cometary ions take up the role of the light solar wind ions in the plasma flow around a comet (Williamson et al., 2020). The magnetic field and electron fluid continue together with the cometary ion fluid toward the nucleus, where a diamagnetic cavity is formed. The extension of the cavity is variable depending on input conditions and it fluctuates heavily, illustrated by positions (a) and (b) in Figure 9.

In the special case of ${\vec{v}}_{\text{sw}}\|{\vec{B}}_{\text{IMF}}$, the convective electric field is zero and therefore cometary ions are only interacting with the solar wind fluid via wave‐particle interactions, which is less efficient than convective electric field pick‐up. The solar wind is therefore not substituted in the ion flow and can penetrate into the inner coma. There, the interplay of gyroradius effects, the presence of an ambipolar field, and the incoming proton velocity allows for protons to be observed inside the diamagnetic cavity in the case where ${\vec{v}}_{\text{sw}}\|{\vec{B}}_{\text{IMF}}$. There is some evidence from previous simulation work that the configuration of ${\vec{v}}_{\text{sw}}\|{\vec{B}}_{\text{IMF}}$ can lead to an expansion of the cavity (from positions (c) to (d) in Figure 9), which is necessary for Rosetta to be able to observe it at the time of the cases presented here.

Whereas several questions remain this hypothesis is now the most likely. For further tests and insights, numerical simulations or multi‐spacecraft observations are necessary and are left for future investigations. Simulations with the Hybrid‐kinetic AMITIS code by Fatemi et al. (2017) have been performed and show good agreement with the observations. A publication is in preparation.

## Figures and Tables

**Figure A1:**
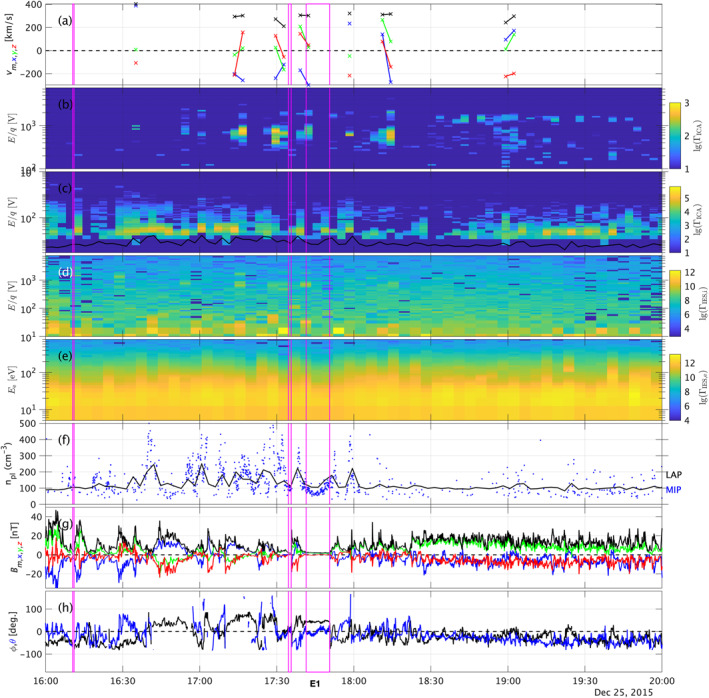
Observations for E1. From top to bottom: (a) solar wind moments, (b) solar wind energy spectra (ICA differential flux averaged over all angles, cm^(−2)^s^(−1)^sr^(−1)^), (c) heavy ion energy spectra (ICA, same units as (b)) and the negative of the spacecraft potential as a black line, (d) ion energy spectra (IES), (e) electron energy spectra and the summed flux as a white line, (f) plasma density from LAP and MIP, (g) magnetic field, and (h) magnetic field cone and clock angle. The magenta lines indicate the interval when Rosetta was in the diamagnetic cavity.

**Figure A2:**
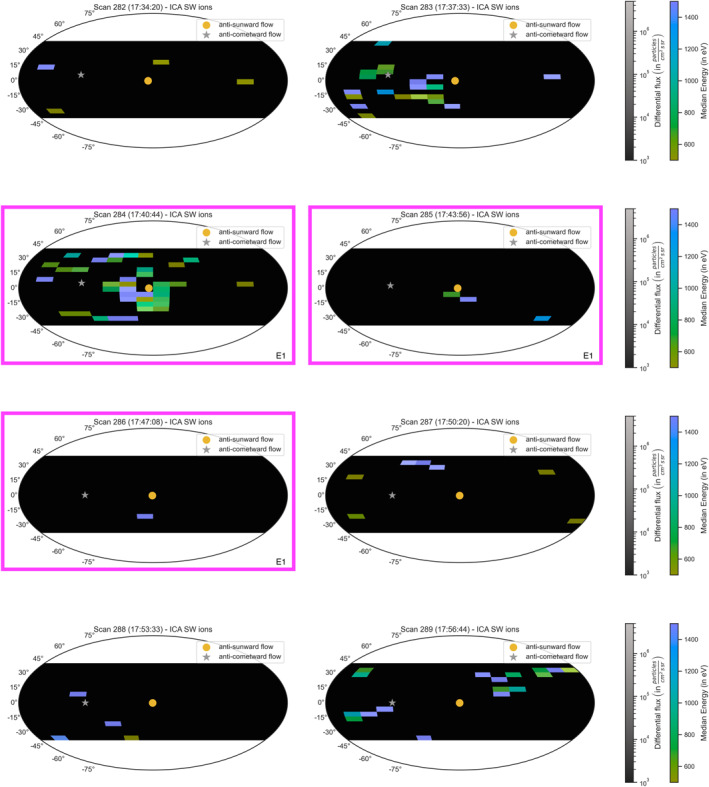
Elevation‐azimuth resolved ICA solar wind spectra for E1. The color gives the mean energy in each bin and the brightness indicates the differential flux. The magenta boxes indicate which spectra are associated with the diamagnetic cavity. The coordinate system is that of the ICA field of view.

**Figure A5:**
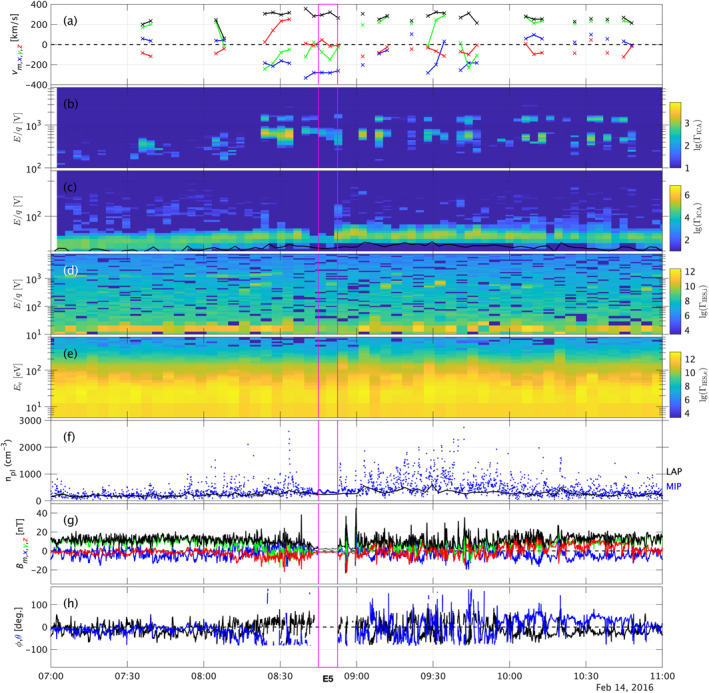
Observations for E5. From top to bottom: (a) solar wind moments, (b) solar wind energy spectra (ICA differential flux averaged over all angles, cm^(−2)^s^(−1)^sr^(−1)^eV^(−1)^), (c) heavy ion energy spectra (ICA, same units as (b)) and the negative of the spacecraft potential as a black line, (d) ion energy spectra (IES), (e) electron energy spectra and the summed flux as a white line, (f) plasma density from LAP and MIP, (g) magnetic field, and (h) magnetic field cone and clock angle. The magenta lines indicate the interval when Rosetta was in the diamagnetic cavity.

**Figure A6:**
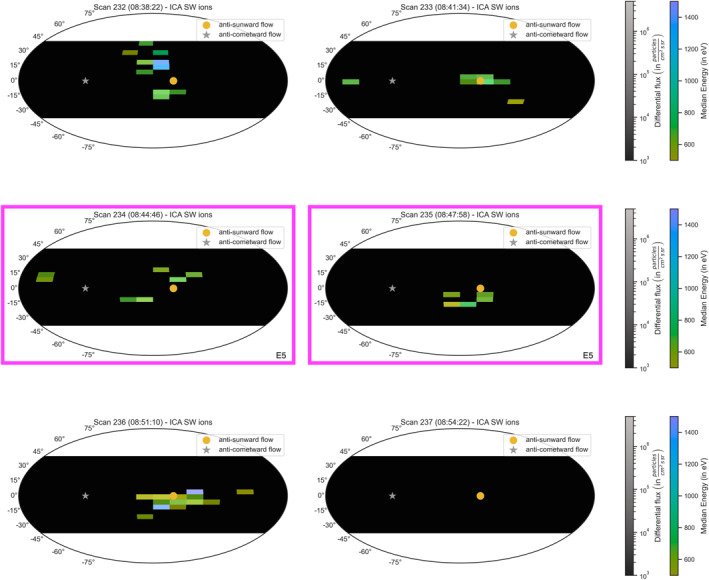
Elevation‐azimuth resolved ICA solar wind spectra for E5. The color gives the mean energy in each bin and the brightness indicates the differential flux. The magenta boxes indicate which spectra are associated with the diamagnetic cavity. The coordinate system is that of the ICA field of view.

**Figure A10:**
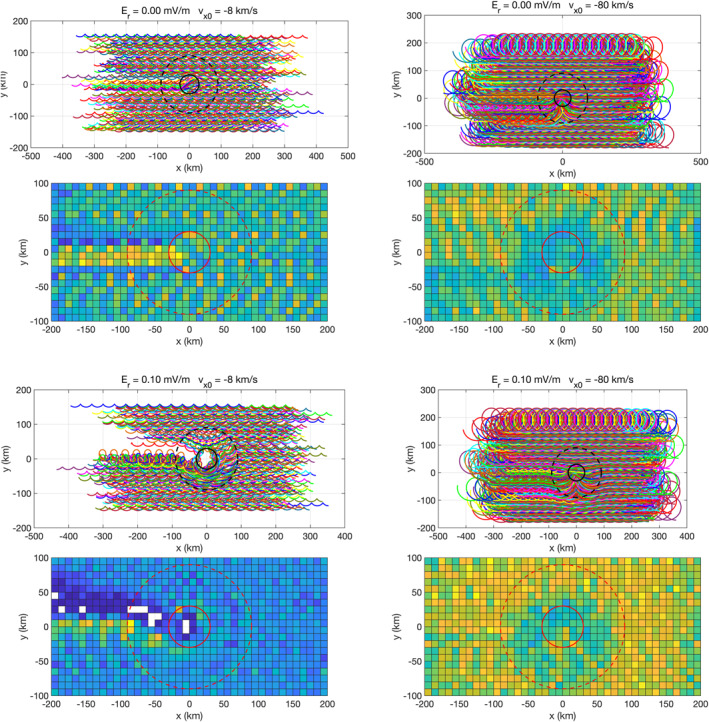
Trajectories and number densities of the protons in the simple model. We show four cases: low velocity (left column), high velocity (right column), zero ambipolar electric field (top row), and nonzero ambipolar electric field (bottom row). The diamagnetic cavity boundary is shown as a solid line and the extent of the ambipolar electric field is marked by a dashed line.

## Data Availability

The RPC and Rosina data used in the study are freely available at the Planetary Science Archive (PSA) via psa.esa.int (Besse et al., 2018). RPC‐MAG data are the Version 9.0 data set last updated May 2020. RPC‐ICA data are level 4 (level 5 for moments) data, last updated July 2021. RPC‐IES data are level 3, version 1 data last updated September 2017. RPC‐LAP and RPC‐MIP data (cross calibrated) are level 5 last updated December 2020. ROSINA‐COPS data are level 2 last updated July 2019. The OMNI data set is freely available at https://omniweb.gsfc.nasa.gov.

## Appendix A Context and Additional Measurements

The high time resolution spectra shown in Figures 1, 2, 3 are advantageous to distinguish shorter time scale variation and ascertain the unambiguous presence of protons in the diamagnetic cavity. In addition, we show context and angle resolved spectra of the observations below. We also compute the cone *ϕ* and clock *θ* angle of the magnetic field:

(A1)
$$\tan\left(\phi \right)=\frac{B_{x}}{\sqrt{B_{y}^{2}+B_{z}^{2}}},\;\tan\left(\theta \right)=\frac{B_{z}}{B_{y}}$$

where *B*_*x*_, *B*_*y*_, and *B*_*z*_ are the three components of the magnetic field (Figure A1).

### A1 E1: 25 December 2015

The solar wind (panels a and b) in the entire interval is already quite deflected, but not slowed down significantly, especially at the beginning. There is a weak proton signature visible in the IES measurements (panel d) in the spectrum recorded around the crossing into the diamagnetic cavity. This is consistent with the partly overlapping field of views of IES and ICA. The electron flux (panel e) is unremarkable and does not change during this interval. The electron density (panel f) is showing large‐scale variations in the density in the first half of the interval. This is in accordance with the behavior of the electron density close to the diamagnetic cavity (Henri et al., 2017). Shortly after 18:00, the density decreases to about 100 cm^−3^ and becomes much more stable. In the diamagnetic cavity (magenta box), the density is relatively low and the electron flux is slightly decreased in accordance with previous studies (Madanian et al., 2017). Thus, we can identify three plasma regions here: First Rosetta measures the highly dynamic and erratic plasma dominated by large‐scale wave structures, i.e., typical for the region just outside the diamagnetic cavity. A new observation is that the solar wind is also penetrating this part of the plasma with very large deflection angles. Then Rosetta enters the diamagnetic cavity, where the field is close to zero, and all large‐scale plasma variations cease. Then Rosetta reenters the first region briefly, before transitioning to the third region. There the plasma is quieter, and the solar wind is less deflected.

Figure A2 shows the angular distribution of the solar wind ions for this event. As discussed above, the solar wind ions are very faint. The first and last spectrum show ion distributions typical of the inner coma of an intermediately active comet: low to no ion fluxes which are deflected away from the solar wind direction. However, in the spectra inside the diamagnetic cavity and those just before and after it, the ions are more antisunward than usual and there are very clear signatures of solar wind particles inside the diamagnetic cavity. This supports our observations in the main text.

### A2 E2–E4: 31 January 2016

The plasma for these events behaves very similar to E1. In addition, the plasma density in the diamagnetic cavity is higher than it is outside, this is probably due to neutral gas density variations. The quiet plasma that was observable at the end of the interval shown for E1 cannot be observed near events E2–E4 at all (Figure A3).

Figure A4 shows the angular distribution of the solar wind ions for this event. The angular distribution of the ions is less clear than for event E1 but there is some indication again that the protons measured in the diamagnetic cavity are more antisunward than those outside. For event E4, the spectrum immediately after the event also shows more antisunward protons but with more spread. This is in general agreement with the moments presented in the main text.

### A3 E5: 14 February 2016

The plasma for these events behaves very similar to the other events. The quieter plasma and magnetic field that was also observed after E1, can be observed again, this time from the start of the interval up to about 8:30 and then again at the very end of the interval. This also corresponds to the lower plasma densities in the quiet region compared to the interval directly after E5 where the density is more variable and higher, and the magnetic field is characterized by steepened wave structures (Ostaszewski et al., 2020). At 8:20, there is a change in the solar wind spectra, which go from broad and low energy to higher energies and more focused. Helium appears in the field of view and the spacecraft enters the higher density, higher field variability region. This kind of transition has been observed often, and was identified as an infant bow shock crossing (Goetz et al., 2021; Gunell et al., 2018) (Figure A5).

Figure A6 shows the angular distribution of the solar wind ions for this event. The angular distributions of the ions again show a highly deflected solar wind especially in the spectra after event E5. The protons inside the cavity are spread out, but more antisunward than those outside. The spectrum before the event already shows some more antisunward motion. This agrees with the moments discussed above and in the main text.

### A4 Comparison Event

To illustrate the uniqueness of the observations during E1–E5, Figure A7 shows observations for a similar interval where the diamagnetic cavity was detected with protons outside of the cavity, but not inside. Note that there is technically one point where there are protons in the cavity but that spectrum is actually half outside and therefore the spectra are not uniquely taken inside the cavity. High time resolution shows that the protons are detected outside the cavity. The difference is that the protons outside of the cavity are much more deflected than they are for events E1–E5.

### A5 Additional Results From the Charge‐Exchange Model

Figures A8 and Figure A9 show the results of the charge‐exchange model for events E2–E5 analogous to Figure 8. The fluxes were calculated as described in Section 3.2.3.

### A6 Simple Ion Trajectory Model

In order to get a better understanding of the ion kinetics near the diamagnetic cavity, we have developed a simple model to calculate the ion trajectories in the inner coma, specifically near the diamagnetic cavity. Within this model, we calculate the Lorentz force on an ion in a given magnetic and electric field environment in 2D. The ion trajectories are solved with a simple Runge‐Kutta method. The diamagnetic cavity is defined as a sphere with zero field around the origin, and we also introduce an outward pointing ambipolar electric field as was done by Vigren and Eriksson (2017). We describe the situation with an electric and magnetic field defined as:

(A2)
$$E\left(r\right)=E_{r}\frac{\tanh\left(-r+3r_{\mathrm{cav}}\right)+1}{2}$$

(A3)
$$B\left(r\right)=B_{z}\frac{\tanh\left(r-r_{\mathrm{cav}}\right)+1}{2}$$

with the cavity radius *r*_*cav*_ = 30 km and *B*_*z*_ = 20 nT. The magnetic field is in the direction perpendicular to the modeled plane. The electric field was chosen to be radial and effective in the spherical region around the origin three times the size of the cavity for computational convenience. The precise shape of the profile is not of importance for the result, as the influence of the electric field is integrated over the trajectory of the ion and therefore a field that decreases linearly (instead of in a step function as is implemented here) has a similar effect on the ion trajectory. We chose to use a value of *E*_*r*_ = 0.1 mV/m as was done by Vigren and Eriksson (2017). In addition, we choose a constant background bulk flow velocity of 10 km/s in accordance with velocity observations. This is the velocity at which the magnetic field moves. This value was chosen because even though the observed solar wind ions are faster, the newborn cometary ions are still present and slow and will give smaller bulk velocities. We then insert protons at 250 ± 50 km in front of the nucleus with a given initial velocity (8 km or 80 km). This gives a good coverage for the ions in the entire simulation domain. Figure A10 shows the result of that model. The implications are discussed in the main text (Figure A10).
